# Properties of Ni and Ni–Fe nanowires electrochemically deposited into a porous alumina template

**DOI:** 10.3762/bjnano.7.163

**Published:** 2016-11-14

**Authors:** Alla I Vorobjova, Dmitry L Shimanovich, Kazimir I Yanushkevich, Sergej L Prischepa, Elena A Outkina

**Affiliations:** 1 Belarusian State University of Informatics and Radioelectronics, P. Brovka 6, Minsk 220013, Belarus; 2 Scientific and Practical Materials Research Center, Institute of Semiconductor and Solid State Physics, National Academy of Sciences of Belarus, P. Brovka 19, Minsk 220072, Belarus

**Keywords:** electrochemical deposition, nanowire, porous alumina template, specific magnetization

## Abstract

The comparative analysis of the electrochemical deposition of Ni and Ni–Fe nanowires (NWs) into ordered porous alumina templates is presented. The method developed allows for obtaining NWs of 50 ± 5 nm in diameter and 25 μm in length, i.e., with an aspect ratio of 500. XRD data demonstrate the polycrystalline nature of Ni and Ni–Fe in a face-centered cubic close-packed lattice. Both fabricated materials, Ni and Ni–Fe, have shown ferromagnetic properties. The specific magnetization value of Ni–Fe NWs in the alumina template is higher than that of the Ni sample and bulk Ni, also the Curie temperature of the Ni–Fe sample (790 K) is higher than that of the Ni sample one or bulk Ni.

## Introduction

Arrays of vertically arranged metallic NWs have attracted a lot of attention due to their shape anisotropy and extremely large surface area. The combination of this unique structure with uncommon magnetic, optical and transport properties can be used to develop novel functional nanomaterials for magnetic, electronic, biomedical and optical nano-scale devices [1–5]. Additionally, the magnetic composite nanostructures are interesting as materials for basic research of magnetic and transport properties in magnetic nanosystems as they possess unique physicochemical properties compared with thin-film and bulk analogues [6–7].

There are different methods to fabricate NWs including electrochemical deposition into porous alumina (PA) templates. The advantages of this method such as low cost, simplicity and efficient testability make it very attractive from the practical point of view [8–10]. Applying of porous alumina as a template allows the formation of vertically ordered NW arrays with uniform geometrical parameters (diameter, length, and density) which can be readily controlled over a wide range of sizes [11–14]. Additionally, porous alumina is a heat-resistant material that allows one to carry out various experiments at high temperature [15–16].

However, preliminary investigations [17–18] have shown that all modes of electrochemical deposition into PA templates have problems such as the blocking of pores by hydrogen bubbles (especially in the case of templates with high aspect ratio) [19]. During deposition in the alternating current mode (ac-deposition) [2,19–20] and pulsed electro-deposition with pauses between the opposite polarity pulses [21–23] etching of the pore walls during the cathodic polarity stage and during pauses occurs. It changes and damages the morphology and smoothness of pore surface and, thus, the reproduction quality and uniformity of the NWs.

The deposition at a certain constant potential (potentiostatic mode, controlled potential electrolysis) is the most commonly used method to fabricate multilayered small-height NWs with the layer thicknesses up to tens of nanometers. Usually, all the mentioned electrochemical procedures are carried out in three-electrode cells. The deposition at a constant current density (galvanostatic mode, dc*-*deposition) is carried out in simpler two-electrode cells, can be easily controlled and is the most common in industrial development.

However, the problem of pore blocking during deposition into the high aspect ratio template requires optimization of the deposition conditions (current density, temperature, electrolyte composition) and adjustment of the parameters of the template (diameter, pore depth and spacing). Therefore, scientific research in this area is relevant especially from the practical point of view.

The properties of the electrodeposited NWs herein depend on the crystal structure of the deposit [24–26]. This concerns particularly magnetic NWs fabricated in various modes of electrochemical deposition of metals, alloys and multilayer compositions into PA templates [27–29]. Some unique magnetic phenomena, such as GMR effect, magnetic crystalline anisotropy, magneto-optical properties depend directly on morphological characteristics (primarily the aspect ratio) and the crystal structure of the NWs [30–32]. Consequently, the features of the electrochemical deposition of various metals (and their composition) into PA templates are still an urgent research issue.

In this work, the formation of Ni and Ni–Fe NWs by dc electrochemical deposition into PA templates with varying heights is presented. The structural properties of PA/Ni and PA/Ni–Fe NWs, as well as the temperature dependence of the specific magnetization of these nanocomposites are investigated and discussed.

## Experimental

### Preparation of porous alumina template

The preparation technique and the thickness of the PA template substantially define the result of metal electrodeposition. Therefore, in spite of the fact that this procedure became almost standard, the technology for the formation of the PA template is constantly improved by researchers. Generally, before the deposition of NWs, the thick alumina film is detached from the Al substrate after removing the barrier layer at the bottom of the pores. Next, a conductive layer is formed by means of sputtering a metal usually onto the back side of the template with continuous nanochannels [2,19,33–34].

In our work, the custom-made PAs were prepared by dc anodization of Al foil, as described in details elsewhere [35]. First, commercial aluminium foil (99.995%) with a size of 60 × 48 mm and a thickness of ca. 100 µm is annealed at 350 °C for 1 h. Then, the samples were electropolished in a mixture of chloric acid and acetic acid 1:4 (volumetric ratio) at *T* ≈ 8 °C and a voltage of 25 ± 2 V for 1–2 min to reduce the surface roughness. Next, the samples were washed in distilled water and dried in a dry air stream. Before anodization, the technological frame has been formed along the perimeter and in the center of the substrate. It is necessary to strengthen the mechanical stability of a free-standing membrane and to restrict certain zones with identical surface area. The frame destination and its formation procedure are described in more detail in [35]. Thick porous alumina films with ordered structure of pores have been prepared by two-step anodization in aqueous solution of oxalic acid (H_2_C_2_O_4_, 0.3 M) at 15 °C. The first stage of anodization was performed under a constant voltage of 50 ± 5 V for 25 min. After the first anodization, the preformed oxide film was removed by wet chemical etching in a mixture of phosphoric acid (H_3_PO_4_, 0.5 M) and chromic acid (H_2_Cr_2_O_7_, 0.2 M) at 80 ± 5 °C for 5 min. The second stage of anodization was performed under the same conditions for 1 to 4 h.

**Figure 1:**
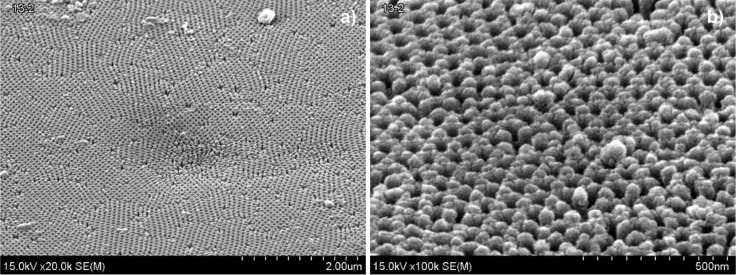
SEM top view of the obtained porous alumina oxide at different magnification a) 20000×, b) 100000×.

Then, electrochemical etching of the barrier layer at the bottom of the pores was carried out by gradual reduction of the forming voltage down to 15 ± 2 V*.* Further, the detachment of alumina from the substrate was performed by Al dissolution in a saturated solution of hydrochloric acid and cupric chloride (HCl + CuCl_2_). Chemical dissolution of the rest of a barrier layer at the pore bottom and chemical pore widening was performed in 4 wt % Н_3_РО_4_ (30 °C) for 15 min. Finally, an electric contact metal (Ta 300 nm + Ni 300 nm or Ta 300 nm + Cu 300 nm) layer was sputtered onto the back side of PA, and a protective coating of chemically resistant varnish HSL (perchlorovinyl lacquer) was applied. As a result, the alumina template with a 30–90 µm thick ordered structure (Figure 1) with pore diameters of 50 ± 5 nm has been fabricated.

### Galvanostatic dc deposition

We used galvanostatic dc deposition to synthesize Ni and Ni-Fe NW arrays in the PA template. A current density in the range of 10 to 50 mA·cm^−2^ is generally used for electroplating Ni onto the flat surface of various substrates. When deposition is carried out on a porous template, the metal is deposited only at the bottom of the pores the effective surface area of which is smaller than the visible surface area. At a pore density of 10^10^ cm^−2^ and pore diameter of 50 nm the space occupied by the bottom of the pores on a surface of 1 cm^2^ is equal to about 0.2 cm^2^. For example, a current density of 15 mA·cm^−2^ corresponds to an effective current density of about 3 mA·cm^−2^ for electrodeposition on a porous template.

It has been found experimentally that, by applying a low current density of 3.0 mA·cm^−2^ during galvanostatic dc deposition on a porous template, uniform, highly ordered, densely packed NWs of about 25 micrometers length are formed. Similar data have been described in [2] for the synthesis of Co NWs in an oxalic acid alumina template.

The solution for Ni–Fe NWs electrodeposition was prepared using NiSO_4_·7H_2_O and FeSO_4_·6H_2_O as sources of Ni and Fe ions, and H_3_BO_3_ as a stabilizer. To fabricate Ni NWs, we used a solution containing NiSO_4_·6H_2_O and NiCl_2_·6H_2_O as a nickel source, and boric acid as a stabilizer. NaOH was used to adjust the pH value of the solution (pH meter HI83141, HANNA instruments). Preventers (Na_2_SO_4_, CuSO_4_) were added to decrease corrosion activity of the electrolyte. This is particularly important during long-term deposition experiments. The concentration and pH value of each solution are shown in Table 1.

**Table 1:** Characteristics of electrolyte solutions used for galvanostatic dc electrodeposition of Ni and Ni–Fe.

electrolyte solution	pH	compounds	concentration, g·L^−1^	function

Ni (no.1)	5.2	NiSO_4_·6H_2_O	140	nickel source
		NiCl_2_·6H_2_O	30	nickel source
		H_3_BO_3_	25	stabilizer
		Na_2_SO_4_	6	preventer
		NaOH		to adjust pH
Ni–Fe (no. 2)	3.0–3.5	NiSO_4_·7H_2_O	90	nickel source
		FeSO_4_·6H_2_O	13.5	iron source
		Н_3_BO_3_	25	stabilizer
		CuSO_4_	2	preventer
		NaOH		to adjust pH

All experiments were performed at room temperature (22 ± 2 °C) using 3.0 cm^2^ area samples at a constant current density of 3.0 mA·cm^−2^ and varying deposition times from 10 to 150 min. The alumina thickness was varied in the range of 30–90 μm. The deposited material mass was determined using a micro-analytical electronic balance (Sartorius CP 225D, accuracy of 0.01 mg). The templates were weighed before and after the metal deposition. All the electrochemical processes (anodizing and deposition) were carried out in a two-electrode cell. A graphite plate was used as auxiliary electrode. The electrochemical processes parameters were controlled with the electronically measuring galvanostatic/potentiostatic power supply P-5827M (potentiostat).

The morphology of PA/Ni and PA/Ni–Fe nanocomposites formed with different alumina template thicknesses and deposition durations was studied by scanning electron microscopy (Philips XL 30 S FEG). The crystal structure of the NWs was studied using X-ray diffraction (DRON-3M diffractometer) with Cu Kα radiation (λ = 1.54242 Å).

To measure the specific magnetization σ as a function of the temperature in the range of 77–1400 K, the ponderomotive method was used [36]. The applied magnetic field was 860 mT, and the measurements precision for σ was ±0.01 A·m^2^·kg^−1^. More details about this method and used equipment can be found elsewhere [37].

## Results and Discussion

**Figure 2:**
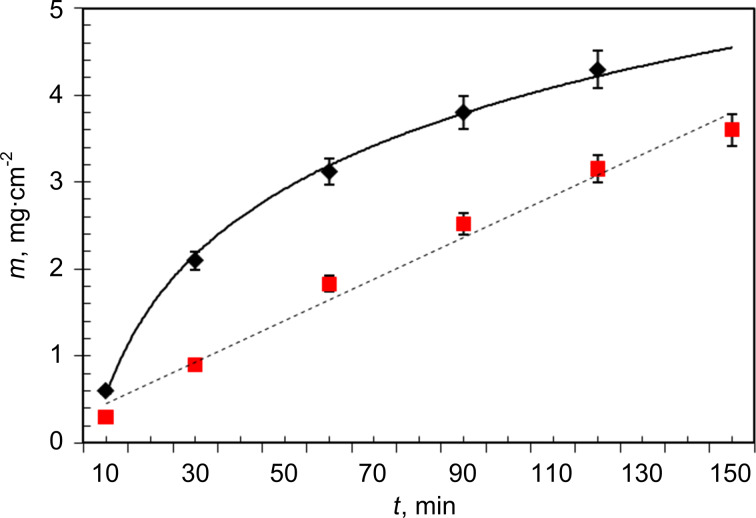
Deposited mass of Ni and of Ni–Fe as a function of the deposition time. Alumina thickness (*H*_PA_) is 45 μm; pore diameter is 50 ± 5 nm. Black diamonds: Ni from solution no. 1; red squares: Ni–Fe from solution no.2.

The dependence of the Ni–Fe mass (*m*_Ni–Fe_) on the deposition duration compared to *m*_Ni_ deposited under the same conditions is shown in Figure 2. Apparently, the amount of deposited Ni–Fe increases almost linearly over the 120 min in contrast to Ni the growth rate of which gradually decreases over the deposition time.

**Figure 3:**
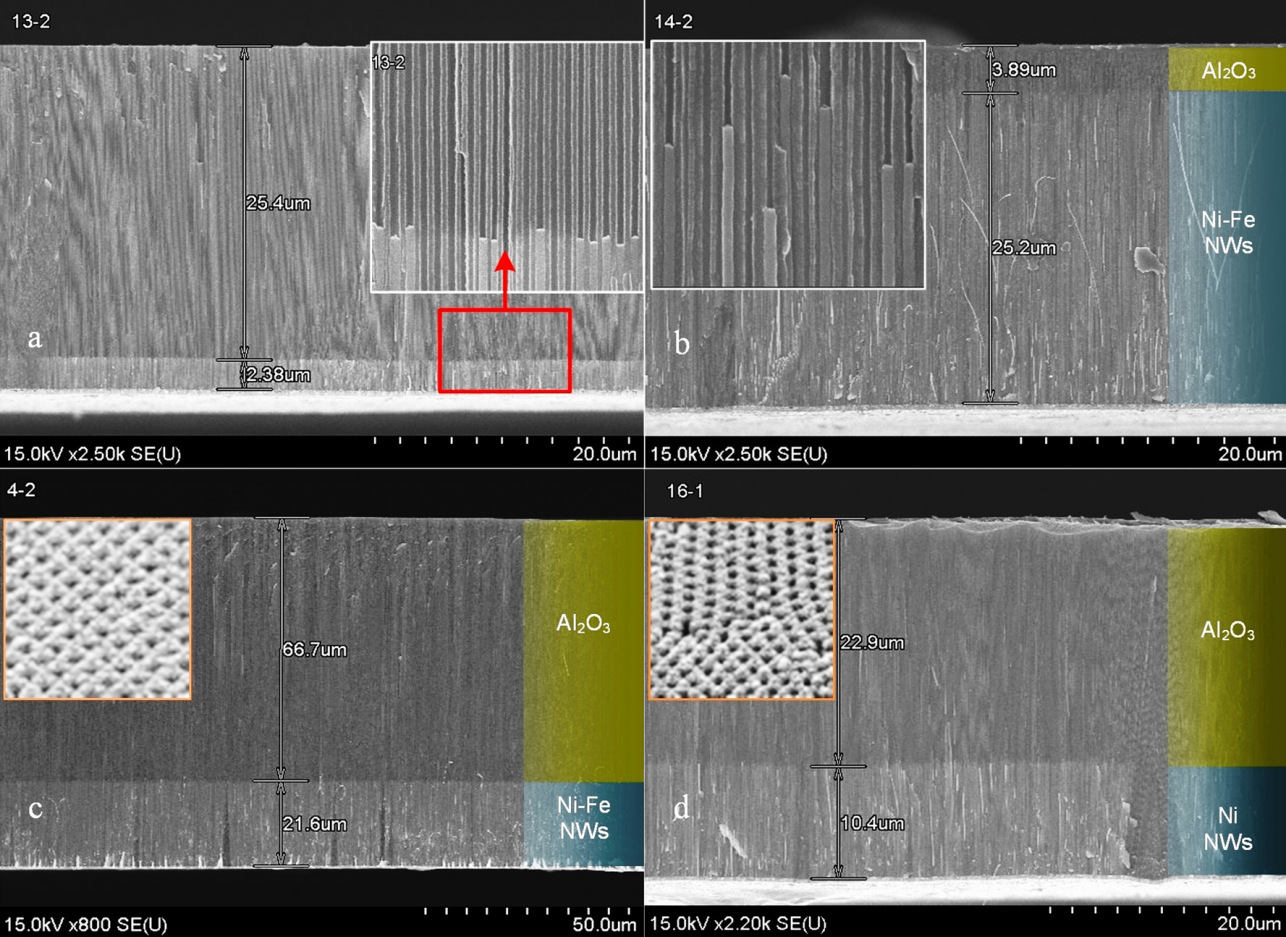
SEM cross-sectional views of the alumina template after galvanostatic dc deposition of Ni–Fe (a, b, c) and Ni (d) NW arrays under the following conditions: current density 3 mA·cm^−2^, deposition duration 10 min (a, d), 90 min (b) and 150 min (c). In the insets: the top-view SEM images of the template (red frame) and the enlarged image of the deposition front depending on deposition duration.

In Figure 3, the SEM cross-sectional views of PA/Ni–Fe (a–c) and PA/Ni (d) composites prepared at the same current density (3 mA·cm^−2^) for 10, 90, 150 min (a–c) and 10 min (d) and for varying thicknesses (*H*_PA_) of the alumina template (ca. 30 μm in panels a, b and d, ca. 90 μm in panel c) are presented.

The morphology of the deposition boundary of NW arrays deposited into alumina for a short duration is presented in the inset of Figure 3a. In this case, Ni–Fe NWs of 2.0 to 2.5 μm length are ordered and uniform. From the photos it is evident that at the same thickness of the template (ca. 30 μm) the deposition front changes slightly with increasing deposition time. In this case (*H*_PA_ ≈ 30 μm), the filling rate *v*_Ni–Fe_ is about 15 μm/h (Figure 3a,b). For the alumina template with H_PA_ ≈ 90 μm the filling rate *v*_Ni–Fe_ is about 8.6 μm/h (Figure 3c) at the same current density of 3 mA·cm^−2^. There are two possible reasons for the lowering of the filling rate: (i) the movement of liquid (electrolyte) in long narrow pores becomes difficult, and the quantity of ions that is necessary to maintain deposition process decreases, i.e., the template resistance increases; (ii) irrespective of synthesis conditions in the course of electrochemical reaction, hydrogen is generated and hydrogen bubbles block the pores of the PA template.

From Figure 3 it is also evident that NWs fill each of the pores. Both pores and NWs have smooth and straight edges; the diameter of a NW is equal to the diameter of the pore. It should be noted that the cross-sectional SEM images were obtained by cleaving the PA, thereby the detachment of some NWs from their pores occured. After increasing the deposition duration (with other parameters being equal) the length of the NWs increases almost linearly to 25 μm, which corresponds to an aspect ratio of 500. The parameters of the galvanostatic dc deposition of Ni and Ni–Fe at a current density of 3 mA·cm^−2^ and varying *H*_PA_ are summarized in Table 2.

**Table 2:** The results of the galvanostatic dc deposition of Ni and Ni–Fe at a current density of 3 mA·cm^-2^ and varying H_PA_.

no.	type	*H*_PA_, µm	*H*_Me_, µm	*t*, min	*m*_Me_, mg/cm^2^	*v*_Me_, μm/min	*v_m_*, mg·cm^−2^s^−1^

1	Ni	33.3	10.4	10	0.60	1.04	0.060
2	Ni	40	—	60	3.12	—	0.052
3	Ni	65	—	120	4.29	—	0.036

4	Ni–Fe	27.8	2.4	10	0.30	0.24	0.030
5	Ni–Fe	50	—	60	1.83	—	0.028
6	Ni–Fe	29.1	25.2	90	2.52	0.28	0.028
7	Ni–Fe	65	—	90	2.56	—	0.028
8	Ni–Fe	88.3	21.6	150	2.67	0.14	0.018

The growth rate *v*_m_ for Ni–Fe NWs is half of that of Ni NWs with all other parameters being equal (line 1 and 4 in Table 2). The dependence of the growth rate on the oxide thickness for Ni NWs is more obvious. In the case of Ni–Fe NWs, the growth rate is almost independent of *H*_PA_ in the range of 30–65 µm and decreases only at *H*_PA_ ≈ 90 µm (lines 5–7 of Table 2). The obtained results and the scanning electron microscopy data show that the quality of the NWs (smoothness, thickness homogeneity, continuity) depends on the evenness of the deposition process as well as on the perfection of a template. The evenness of the deposition process, in turn, depends on the pore filling rate and, partly, on the template thickness, especially in the case of Ni.

**Figure 4:**
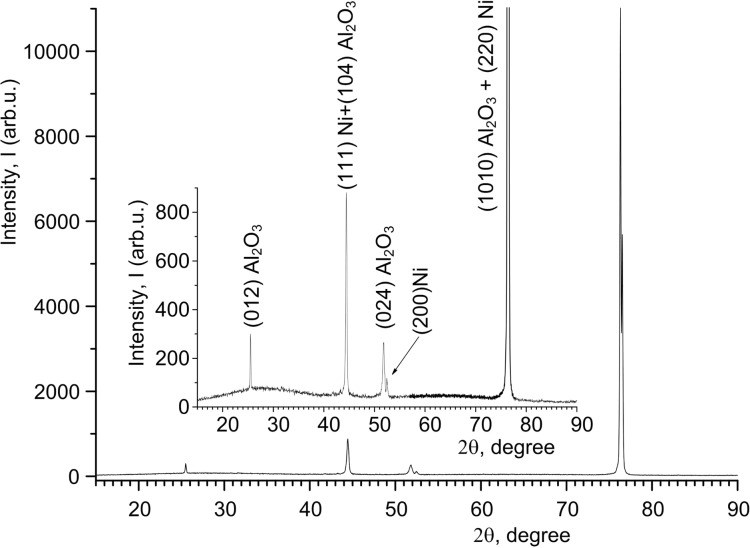
XRD pattern for Ni NWs in alumina template with *H*_PA_ of 65 μm and a current density of 3 mA·cm^−2^, deposition duration 120 min.

**Figure 5:**
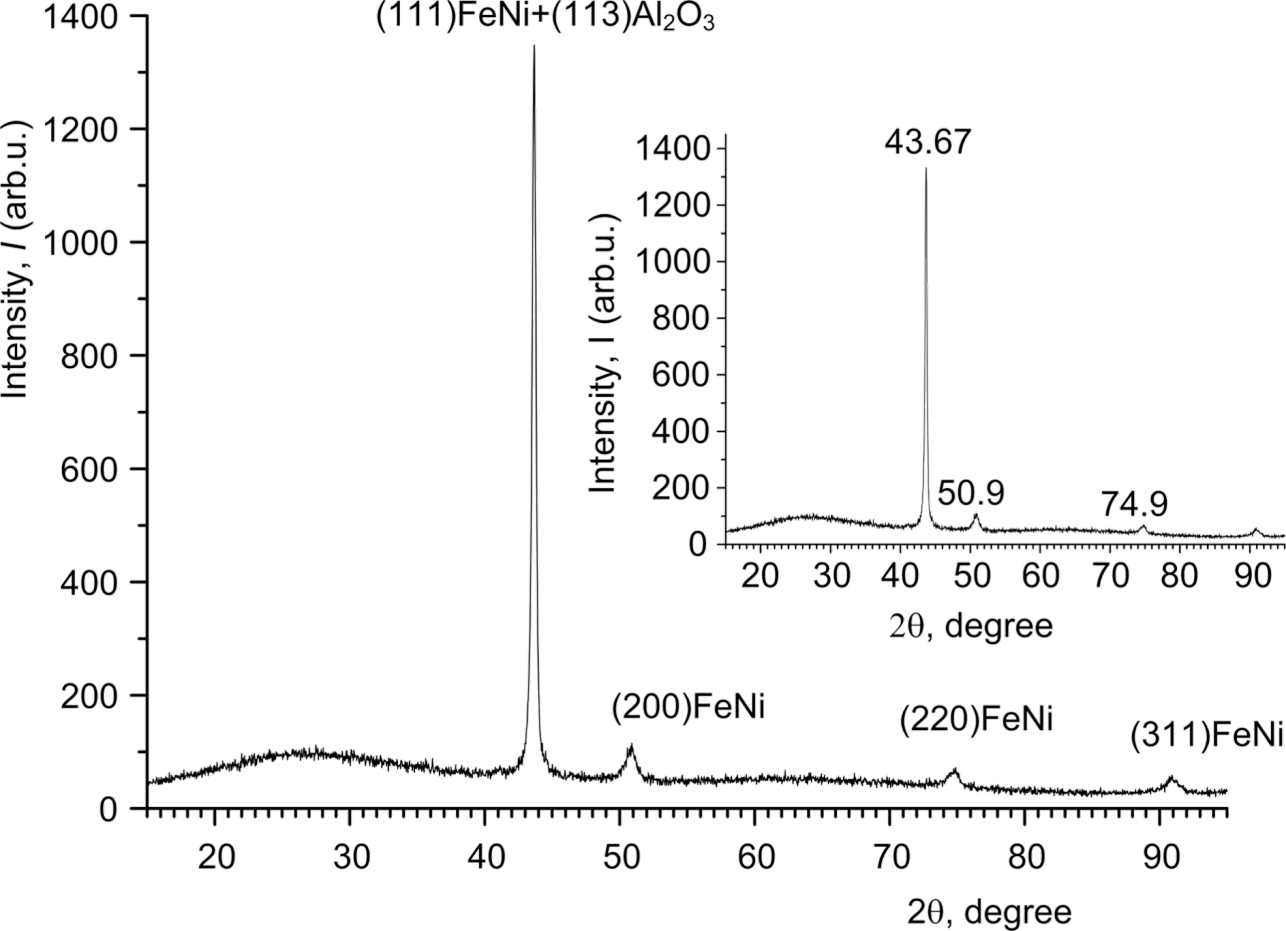
XRD pattern of Ni–Fe NWs inside the alumina template with *H*_PA_ of 50 μm and a current density 3 mA·cm^−2^, deposition duration 90 min.

In the following, the results of crystal structure investigations are discussed. The XRD patterns of PA/Ni and PA/Ni–Fe composites are shown in Figure 4 and Figure 5, and summarized in Table 3.

**Table 3:** The main characteristics of XRD patterns for Ni and Ni-Fe in Al_2_O_3_.

Ni–Fe				Ni		
no.	crystal orientation	2θ, °	intensity, %	crystal orientation	2θ, °	intensity, %

1	Ni–Fe(111) + Al_2_O_3_	43.67	100	Ni(111) + Al_2_O_3_	44.45	11
2	Ni–Fe(200) + Al_2_O_3_	50.90	29	Ni(200) + Al_2_O_3_	51.76	1.3
3	Ni–Fe(220) + Al_2_O_3_	74.90	9	Ni(220) + Al_2_O_3_	76.53	100
4	Ni–Fe(311) + Al_2_O_3_	90.10	7	Ni(311) + Al_2_O_3_	89.80	0

The character of these XRD data with narrow peaks suggests a crystalline phase in the both cases. The nickel phase (Figure 4) crystallized in a fcc lattice, as evidenced by identification of Ni samples, Table 3, using the ICDD (International Centre for Diffraction Data) reference calculation tables (the Databank PDF).

Besides the main peak of a magnetic phase at 2θ = 76.53° (220) (Figure 4) there are two weaker peaks at 51.76° and 44.45° corresponding to the crystal orientations (200) and (111). As it is reported in [38], these orientations are specific for Ni NWs electrodeposited into alumina template.

The primary direction of Ni growth in alumina pores is the orientation (220), and the height of the corresponding peak is significantly larger than that of other peaks. This demonstrates the high crystallinity of the NWs and the mutual orientation of crystallites along the main direction of growth (the *Z*-axis oriented vertically along a pore). The existence of other weak peaks shows the presence of small amount of crystallites with other directions of growth and confirms the polycrystalline structure of Ni NWs.

Previously it has been shown [39] that for a Ni film with polycrystalline fcc structure the primary direction of crystallite growth is (111) and, thus, it differs from Ni NWs in the alumina pores (220). In our case, the primary direction of Ni NWs growth is (220), too. As described in [40], this orientation is a specific feature of Ni NWs electrodeposited into an alumina template under certain deposition conditions (overpotential and temperature).

Perhaps, the difference in the primary direction of crystallites between a continuous film and NWs is the different behavior of nanocrystallites of the same material deposited on the flat surface (like a film) or into the narrow and long channels (pores) of an amorphous matrix (aluminum oxide) in the form of NWs. The template Al_2_O_3_ is X-ray amorphous (wide peak at 2θ = 25.16°).

The XRD patterns of Ni–Fe NWs also demonstrate the polycrystalline nature of the Ni–Fe phase in the fcc close-packed lattice. The polycrystalline Ni–Fe is deposited with the orientations (111), (200), (220) and (311) (Figure 5). However, for this material the primary direction of NW growth is (111), and the height of the corresponding peak is also larger than that of other peaks. It was reported that under identical conditions of electrochemical deposition the primary orientation of crystallites (texture) for alloy films (Ni–Fe coating) and single-component films (Ni) will be different as a result of significant grain refinement and increasing Fe content in the range from 1 to 25% [41].

The texturing (primary orientation) is caused by a higher binding energy between the co-deposited atoms than between the atoms of a film and a substrate surface [42]. In the alloy case, the co-deposited atoms are different, and the surface area on which they are deposited is limited (bottom of a pore). Besides, the ions transfer process proceeds in narrow prolonged channels. All these features influence the mechanism of crystallization and, therefore, define physical and chemical properties of deposits.

The average size of Ni and Ni–Fe crystallites was calculated using the Debye–Scherer equation:


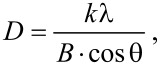


where *D* is the grain size, *k* is equal to 0.90–0.94 for the FWHM (full width at half maximum) of spherical crystals with cubic symmetry, λ is the wavelength of the X-rays (λ = 0.154056 nm in our case), *B* is the FWHM and θ is the half diffraction angle of the peak. Crystallite sizes for Ni and Ni–Fe NWs in the alumina template are presented in Table 4.

**Table 4:** The crystallite sizes for Ni and Ni–Fe NWs in the alumina template.

sample	Ni, NWs				Ni–Fe, NWs			

orientation	111	200	220	311	111	200	220	311
*I*, %	11	1.3	100	—	100	29	9	7
*D*, nm	17.9	18.3	35.3	—	17.9	9.1	6.9	7.8

Further, the investigations of magnetic properties of the obtained composite materials, such as Curie temperature (*T*_C_) and specific magnetization as a function of temperature σ(*T*) have been performed and analyzed. The temperature dependence of σ(T) was studied in the range from 77 to 900 K in the "heating–cooling" mode in a magnetic field of 860 mT, as described in [43]. To avoid oxidation of the metal at high temperatures, the samples were placed into an evacuated ampoule. The magnetic characteristics of the obtained Ni and Ni–Fe NWs are then compared with each other and with those of bulk nickel.

**Figure 6:**
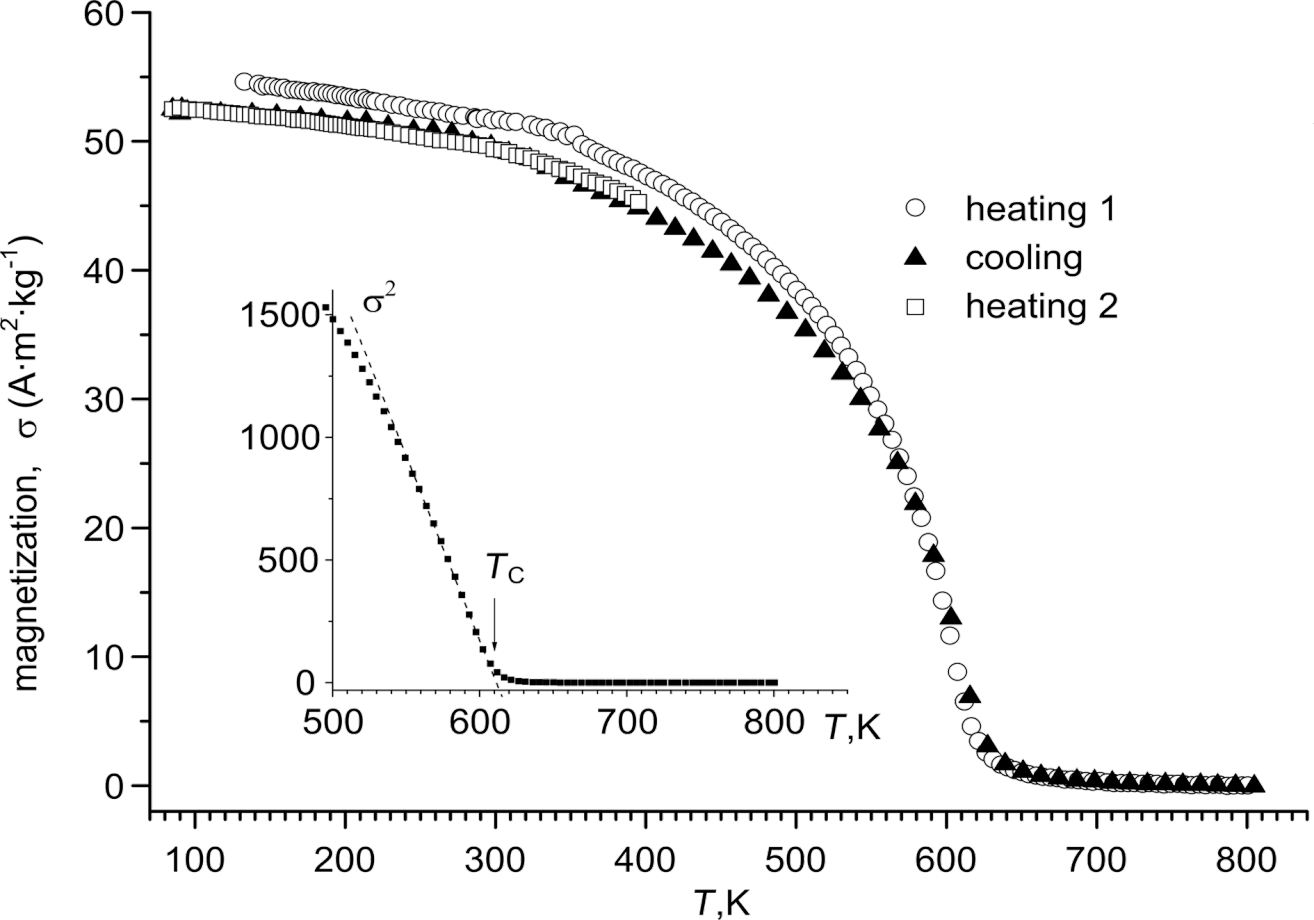
Magnetization as a function of the temperature for Ni NWs fabricated in the alumina template (*H*_PA_ = 65 μm) at a current density of 3 mA·cm^−2^, the deposition duration was 90 min. The inset is the σ^2^(*T*) dependence. The dashed line represents a linear fit to the experimental data. The arrow indicates the evaluated *T*_C_.

**Figure 7:**
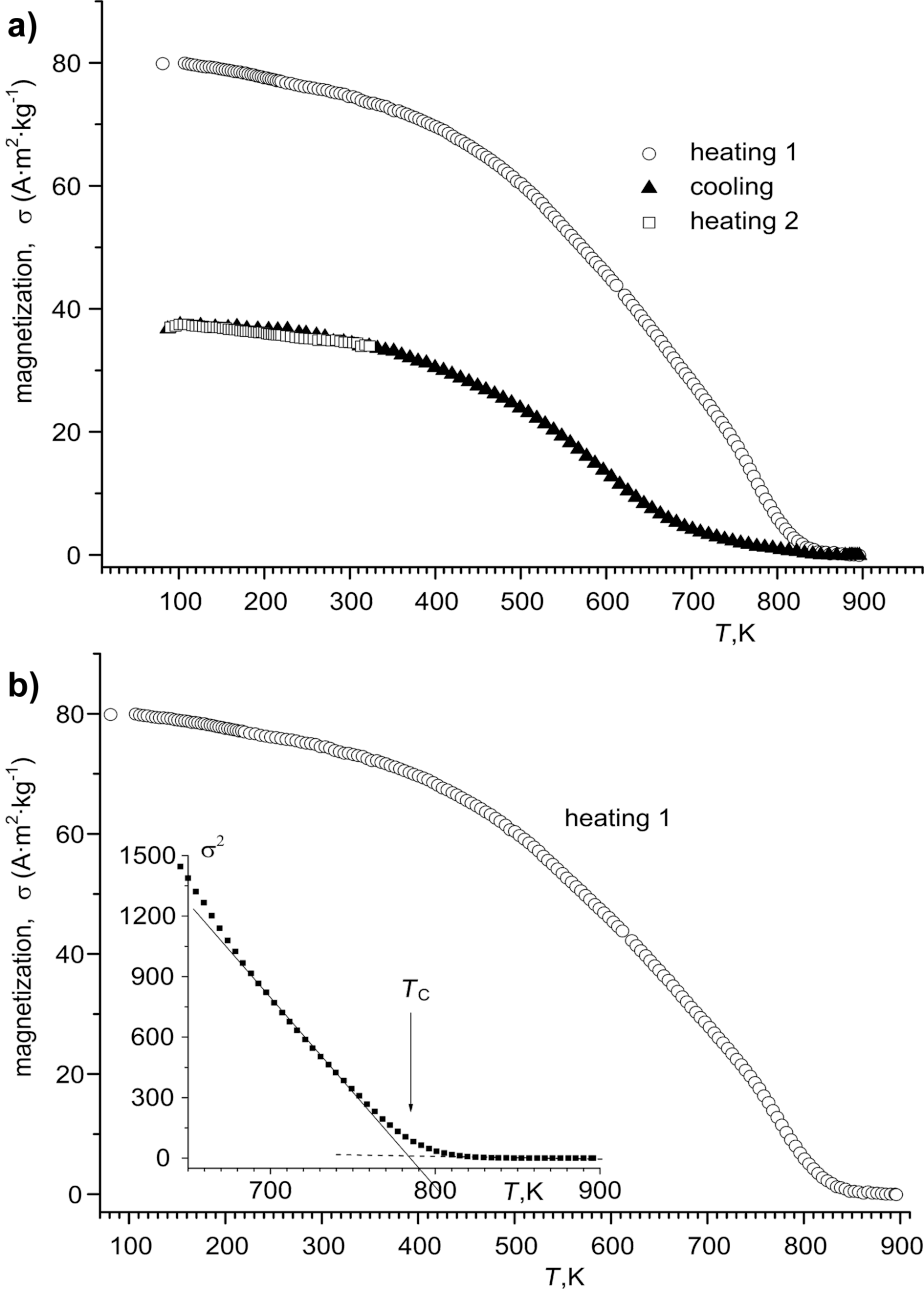
Magnetization as a function of the temperature for Ni–Fe NWs fabricated in the alumina template (a) with *H*_PA_ = 65 μm at a current density of 3 mA·cm^−2^, the deposition duration was 90 min; (b) the same and the inset is the σ^2^(*T*) dependence. The dashed line represents a linear fit to the experimental data. The arrow indicates the evaluated *T*_C_.

In Figure 6, the σ(*T*) dependence of Ni NWs fabricated in the alumina template with *H*_PA_ of 50 μm at a current density of 3 mA·cm^−2^ and a deposition duration of 120 min is presented. The same results for Ni–Fe NWs fabricated in the alumina template with *H*_PA_ of 65 μm at a current density 3 mA·cm^−2^ and a deposition duration of 90 min are presented in Figure 7.

As evident from Figure 6, the specific magnetization value of Ni in the alumina template (54 A·m^2^·kg^−1^ in cooling mode and 56 A·m^2^·kg^−1^ in heating mode) is close to the specific magnetization of bulk Ni (σ ≈ 58.9 A·m^2^·kg^−1^) [44]. For this sample, the cooling behavior is strongly ferromagnetic.

The specific magnetization value of Ni-Fe in the alumina template (80 A·m^2^·kg^−1^, heating mode, Figure 7) is higher than that of the PA/Ni NWs (Figure 6) and bulk Ni and less with respect to permalloy (Py = Fe_20_Ni_80_, σ ≈ 100 A·m^2^·kg^−1^ [45]). The σ(*T*) curve measured in the cooling mode indicates significantly smaller σ values, but the cooling behavior for this sample is also ferromagnetic. The decrease of specific magnetization after heating in vacuum (measuring specificity) could be caused by interaction of the metal with the matrix material of the pores and the formation of a non-ferromagnetic alloy. Similar effect was observed for Ni deposited into porous silicon templates [43,46].

The Curie temperature, *T*_C_, for the fabricated composites was defined according to the Curie–Weiss law, notably, the specific magnetization depends on the temperature as σ ~ (1 − *T*/*T*_C_)^1/2^. In the inset of Figure 6 there is the σ^2^(*T*) dependence for PA/Ni, which allows one to determine the *T*_C_ value as 612 K, which is slightly lower than for bulk Ni (627 K [47]). The *T*_C_ value of the Ni–Fe sample (Figure 7b) is higher than that of the Ni sample and bulk Ni, and is equal to 790 K, which is rather high. Note, that this value is less than the *T*c of bulk Fe (1040 K) and Py (823 K) [47].

## Conclusion

Arrays of compact Ni–Fe NWs with high aspect ratio (about 500) and diameters of 50 nm have been fabricated using a porous alumina template by electrochemical dc deposition at a low current density of 3 mA·cm^−2^. The influence of thickness and structural order of the template on the growth rate and uniformity of deposition process of NWs have been discussed, as these features define the quality of the NWs. The comparative analysis of morphological, structural and magnetic properties between Ni–Fe NWs and Ni NWs and bulk Ni was performed using scanning electron microscopy, X-ray diffraction and custom-built equipment based on the ponderomotive method. The arrays of vertically ordered, straight and smooth polycrystalline Ni NWs with a preferred growth orientation of (220) and Ni–Fe NWs with preferred (111) orientation have shown ferromagnetic properties. The value of specific magnetization of Ni–Fe NWs in the alumina template is higher than that of the Ni sample and bulk Ni. The Curie temperature of Ni–Fe sample (790 K) is higher than that of the Ni sample or bulk Ni, but lower than that of bulk Fe and Py. The possible reasons for the different structural and magnetic properties of single- (Ni) and two-component (Ni–Fe) NWs formed under identical conditions have been discussed. While studying the temperature dependence of the specific magnetization, the possibility for a tuning of the specific magnetization due to interactions of the magnetic material with the matrix material at high temperatures was demonstrated. This effect is more pronounced for Fe–Ni. More work related to the influence of the parameters of bundles of NWs on magnetic properties is in progress now.
